# Differentially Expressed Bone Marrow microRNAs Are Associated With Soluble HLA-G Bone Marrow Levels in Childhood Leukemia

**DOI:** 10.3389/fgene.2022.871972

**Published:** 2022-06-14

**Authors:** Renata Santos Almeida, Thailany Thays Gomes, Felipe Souza Araújo, Sávio Augusto Vieira de Oliveira, Jair Figueredo Santos, Eduardo Antônio Donadi, Norma Lucena-Silva

**Affiliations:** ^1^ Laboratory of Immunogenetics, Department of Immunology, Aggeu Magalhães Institute, Oswaldo Cruz Foundation (Fiocruz), Recife, Brazil; ^2^ Clinical Immunology Division, Department of Medicine, School of Medicine of Ribeirão Preto, University of São Paulo (USP), Ribeirão Preto, Brazil; ^3^ Laboratory of Molecular Biology, Pediatric Oncology Service, IMIP Hospital, Recife, Brazil

**Keywords:** leukemia, HLA-G, microRNA, bone marrow, posttranscriptional regulation, ALL, AML

## Abstract

HLA-G is a nonclassical histocompatibility class I molecule that plays a role in immune vigilance in cancer and infectious diseases. We previously reported that highly soluble HLA-G (sHLA-G) levels in the bone marrow were associated with a high blood cell count in T-acute lymphoblastic leukemia, a marker associated with a poor prognosis. To understand the posttranscriptional *HLA-G* gene regulation in leukemia, we evaluated the bone marrow microRNA profile associated with the HLA-G bone marrow mRNA expression and sHLA-G bone marrow levels in children exhibiting acute leukemia (B-ALL, T-ALL, and AML) using massively parallel sequencing. Ten differentially expressed miRNAs were associated with high sHLA-G bone marrow levels, and four of them (hsa-miR-4516, hsa-miR-486-5p, hsa-miR-4488, and hsa-miR-5096) targeted *HLA-G*, acting at distinct *HLA-G* gene segments. For qPCR validation, these miRNA expression levels (ΔCt) were correlated with *HLA-G5* and *RREB1* mRNA expressions and sHLA-G bone marrow levels according to the leukemia subtype. The hsa-miR-4488 and hsa-miR-5096 expression levels were lower in B-ALL than in AML, while that of hsa-miR-486-5p was lower in T-ALL than in AML. In T-ALL, hsa-miR-5096 correlated positively with *HLA-G5* and negatively with sHLA-G. In addition, hsa-miR-4516 correlated negatively with sHLA-G levels. In AML, hsa-miR-4516 and hsa-miR-4488 correlated positively with *HLA-G5* mRNA, but the *HLA-G5* negatively correlated with sHLA-G. Our findings highlight the need to validate the findings of massively parallel sequencing since the experiment generally uses few individuals, and the same type of leukemia can be molecularly quite variable. We showed that miRNA’s milieu in leukemia’s bone marrow environment varies according to the type of leukemia and that the regulation of sHLA-G expression exerted by the same miRNA may act by a distinct mechanism in different types of leukemia.

## Introduction

HLA-G is a nonclassical MHC class I molecule with particular and distinct characteristics when compared with classical molecules, including restricted tissue expression, little gene variability at the coding region, and significant variability at the regulatory regions. HLA-G exhibits immunomodulatory properties rather than antigen presentation function (Castelli et al., 2014; Carosella et al., 2015; Amodio and Gregori, 2020). Several immune system cell functions, such as the cytotoxic effect of NK and T CD8+ cells, antigen presentation by dendritic cells, among others, are negatively regulated due to HLA-G binding to the inhibitory leukocyte ILT2 (LILRB1), ILT4 (LILRB2), and KIR2DL4 receptors (Colonna et al., 1998; Rajagopalan and Long, 1999; Shiroishia et al., 2003, 2006; Yan and Fan, 2005; Donadi et al., 2011; Rouas-Freiss et al., 2014; Amodio and Gregori, 2020).

*HLA-G* expression has been primarily related to its immunotolerance in pregnancy (Rouas-Freiss et al., 1997; Xu et al., 2020), but differential *HLA-G* levels can also influence the pathogenesis and outcome of infectious and noninfectious diseases (Yan et al., 2009; Rizzo et al., 2008). In cancer, increased HLA-G levels can alter the immunosurveillance mechanism, favoring tumor immune escape (Yan, 2011; Castelli et al., 2014; Rouas-Freiss et al., 2014; Lin and Yan, 2018). High plasma HLA-G (sHLA-G) levels have been associated with immunosuppression and worse prognosis in several hematological malignancies, such as acute and chronic leukemias (Sebti et al., 2003; Gros et al., 2006; Rizzo et al., 2014; Caocci et al., 2017), Hodgkin’s lymphoma (Diepstra et al., 2008; Caocci et al., 2016), and diffuse large B-cell lymphoma (Josionek-Kupnicka et al., 2016).

Little attention has been devoted to the role of bone marrow sHLA-G levels in hematological disorders; however, several lines of evidence indicate its relevant contribution. The sHLA-G levels in the non-leukemic bone marrow are higher than in the peripheral blood (Almeida et al., 2018; Cavalcanti et al., 2017). In a previous study conducted by our group, high bone marrow sHLA-G levels were associated with elevated blood cell count in childhood T-cell acute lymphoblastic leukemia (ALL), a criterion related to poor prognosis (Almeida et al., 2018). Bone marrow sHLA-G levels may be regulated by transcriptional and posttranscriptional factors, which may differentially influence the gene expression depending on the *HLA-G* gene polymorphic sites at regulatory regions and on the microenvironment milieu (Castelli et al., 2010; Castelli et al., 2014; Porto et al., 2015). In this context, differential microRNA expression profiles have been associated with different types of leukemia, such as T-cell ALL (T-ALL) (Schotte et al., 2009; Schotte et al., 2011; Wallaert et al., 2017), B-cell ALL (B-ALL) (Schotte et al., 2009; Schotte et al., 2011), and chronic lymphocytic leukemia (CLL) (Calin et al., 2005), which are targets mainly to genes of innate and adaptive immunity (O’Connell et al., 2010; Mehta and Baltimore, 2016; Omar er al., 2019), particularly genes encoding immune checkpoint molecules (Eichmüller et al., 2017; Hirschberger et al., 2018; Omar et al., 2019).

Since, in T-ALL, only high sHLA-G producers are associated with elevated blood cell count (dos Santos Almeida et al., 2018), this study was designed to clarify the relationship between the sHLA-G levels and the microRNA profiles in the bone marrow of untreated ALL patients to unveil some of the posttranscriptional control of HLA-G in leukemia.

## Materials and Methods

### Study Design, Population, and Ethical Considerations

A group of 15 children with ALL (8 B-ALL and 7 T-ALL) aged between 0 and 18 years were considered for the study of differentially expressed microRNA (DE-miRNA) in bone marrow cells according to the marrow stroma sHLA-G levels. For real-time quantitative PCR validation experiments, we compared the levels of DE-miRNA in another group of ALL patients (23 B-ALL and 11 T-ALL). To demonstrate that the effect observed was related to the lymphoid cell type, we also evaluated samples from 31 children with acute myeloid leukemia (AML). We also included a control group with 14 samples from children whose myelogram confirmed the absence of leukemia. The expressions of the *HLA-G5* and *RREB1* target genes were evaluated in the bone marrow cells of 19 children with B-ALL, 8 with T-ALL, and 28 with AML. All patients were referred, diagnosed, and treated at the IMIP Hospital, Recife, Brazil. Bone marrow aspirates were obtained from each patient at admission and submitted for the isolation of mononuclear cell fractioning for leukemia diagnosis confirmation, performed as previously described (Marques et al., 2011). The samples were stored under –80 °C conditions provided by a laboratory deep freezer, which was protected against power outage by an uninterruptible power supply (UPS) and emergency line. All the patients with leukemia presented at least 70% of blasts in the bone marrow. The samples were obtained after the children’s legal guardians provided informed consent, approving their participation in the study. The study protocol was previously approved by the local ethics committee (CAAE: #13296913.3.0000.5190 and #0073.0.095.000-10). The patients’ (age and sex) and blast (immunophenotype and genetic alterations) features are shown in Table 1.

**TABLE 1:** Characterization of childhood acute leukemia patients.

Features	B-ALL (*n* = 46)	T-ALL (*n* = 16)	AML (*n* = 44)
**Age at diagnosis**			
Minimum	0.3	2.7	0.8
Maximum	15	16	18
Mean	5.7	8.8	9.2
Standard deviation	3.3	3.9	5.2
**Sex**			
Male	28	15	25
Female	18	1	19

**Blast immunophenotype**
	Pro-B	1	ETP	1	AML-M0	6
	Pre-B	40	Pre-T	9	AML-M1	4
	Pre-B	3	Cortical-T	2	AML-M2	11
	Transitional-B	1	Mature-T	4	AML-M3	6
	Mature-B	1			AML-M4	2
					AML-M5	8
					AML-M6	4
					AML-M7	3
**Blast genetic alterations**						
	t (12;21) *ETV6-RUNX1*	5	*SIL/TAL*	1	t (8;21) *RUNX1-RUNX1T1*	5
	t (1;19) *TCF3-PBX1*	2	*HOX11*	0	inv (16) *CBFB/MYH11*	4
	t (9;22) *BCR/ABL*	2	*HOX11L2*	1	t (15;17) *PML-RARA*	4
	t (4;11) *KMT2A-AFF1*	0			t (9;11) *KMT2A-MLLT3*	2
	Negative	37	Negative	14	Negative	29

Note: ETP, Early T-cell precursor.

### Determination of Soluble HLA-G Levels in Bone Marrow

A sandwich ELISA assay was used to measure the soluble HLA-G (shredded HLA-G1 and HLA-G5 isoforms) levels, following the manufacturer’s instructions (BioVendor Laboratory Medicine, Inc., Czech Republic), with the limit of detection of 0.6 Units/mL. Our previous study detected an average of 200 U/mL ± 25 SD (standard deviation) of sHLA-G levels in the bone marrow stroma of healthy children (Almeida et al., 2018). Based on this, patients presenting between 150 and 250 U/mL of sHLA-G levels in the bone marrow stroma, that is, 200 U/mL plus two standard deviations above or below, were defined as the intermediate producers, those presenting with more than 250 U/mL of sHLA-G were high producers, while those who produced less than 150 U/mL were low producers.

### MicroRNA Sequencing Analysis

We used the miRNA sequencing database to evaluate the miRNA expression related to the sHLA-G levels in the marrow stroma. Total RNA extraction, quality assessment, library construction, and miRNA sequencing were performed as described previously (Almeida et al., 2019). miRNA sequencing data have been deposited in the ArrayExpress database at EMBL-EBI (www.ebi.ac.uk/arrayexpress) under the accession number E-MTAB-11621. The sequencing analysis included read quality control and contamination assessment using FastQC (https://www.bioinformatics.babraham.ac.uk/projects/fastqc/) and Cutadapt (Martin, 2011) programs considering a Q-score ≥30 and reads with a length ≥ 17 nucleotides. We used Bowtie (http://bowtie-bio.sourceforge.net/index.shtml) for indexing of human reference genome hg38 version, which is deposited in the UCSC Genome Browser (https://genome.ucsc.edu/). The miRDeep2 2.0.0.8 software (Friedländer et al., 2008) was applied for sequence alignment and miRNA identification, considering miRBase release 21 (http://www.mirbase.org/) (Griffiths-Jones et al., 2006; Kozomara and Griffiths-Jones, 2010). Differentially expressed (DE) miRNA profiles were obtained using the edgeR package (Robinson et al., 2010) and the standard analysis and quantile normalization parameters in the R software (https://cran.r-project.org/), considering at least 20 reads in a minimum of 1 sample, a false discovery rate (FDR) ≤0.05, and a log fold change (logFC) cutoff point of 1 or −1. A comparison of the bone marrow miRNA levels between lower versus higher sHLA-G producers was performed. Target prediction of DE-miRNAs was performed using the miRWalk 2.0 (Dweep et al., 2015), and functional annotation was determined by DAVID tools v.6.7 (Huang et al., 2009; Huang et al., 2009), considering the Kyoto Encyclopedia of Genes and Genomes (KEGG) pathways and Gene Ontology (GO) terms: biological process and FAT level, both with Benjamini–Hochberg (BH)–corrected *p*-values ≤ 0.05. The DE-miRNA alignment with the *HLA-G* gene (NG_029039.1) and mRNA sequences (NM_002127.5) was performed using the RNAhybrid v.2.2 tool (Kruger and Rehmsmeier, 2006), considering the essential features for the interaction of the two molecules, that is, Watson and Crick base pairing, few gaps in the interaction, especially on the seed sequence, seed (2–8 miRNA nucleotide), low free energy (≤−20 Kcal), and interaction with target 3′UTR, coding sequence, and promoter region (Castelli et al., 2014).

A search for genes encoding proteins related to *HLA-G* transcription’s positive and negative regulation was performed, considering previous studies that describe or review the action of such molecules (Moreau et al., 1999; Gobin et al., 2002; Flajollet et al., 2009; Castelli et al., 2014; Yaghi et al., 2016). The positive regulators that were considered were *CREB1*, *CREBBP*, *JUN*, *ATF2*, *IRF1*, *HIF1A*, and *IL10*. The negative regulators that were sought were *RREB1*, *HDAC1*, *CTBP1*, and *CTBP2*. We also considered *REST*, *EHMT1*, *ZEB1*, *ZEB2*, *ZNF217*, and *LSD1* genes since the proteins are members of the CTBP core complex (Shi et al., 2003; Shi et al., 2004) and may exert an indirect influence on *HLA-G* expression. All the positive and negative regulators of *HLA-G* those were considered were analyzed for their ability to interact with the differentially expressed miRNAs in this study, according to the miRTarBase v. 8.0, a database of experimentally validated interactions (Chou et al., 2018).

### MicroRNA Validation by Reverse Transcription Quantitative Polymerase Chain Reaction Assays

For validation experiments, we selected the four miRNAs most likely to target the *HLA-G* gene (NG_029039.1) and messenger RNA (NM_002127.5) based on the sequence alignment analysis (RNAhybrid v.2.2) (Krûger, Rehmsmeier, 2006). The representative scheme showing the interaction site between *HLA-G* and the four miRNAs selected for validation was constructed using the ApE v2.0.61 software (https://jorgensen.biology.utah.edu/wayned/ape/). The TaqMan Advanced miRNA cDNA Synthesis Kit (Life Technologies, Foster City, California, USA), TaqMan Advanced miRNA Assay (reference: miR-191-5p; targets: miR-5096, miR-4516, miR-4488, miR-486-5p; Life Technologies), and TaqMan Fast Advanced Master Mix (Life Technologies) were used according to the manufacturer’s instructions to evaluate the miRNA expression. Reverse transcription PCR (RT-PCR) assays were performed in a SimpliAmp Thermal Cycler (Applied Biosystems, Foster City, California, USA) and quantitative PCR (q-PCR) in a QuantStudio 5 Real-Time System (Applied Biosystems) and 7500 Real-Time System (Applied Biosystems) according to the manufacturer’s instructions.

### Expression of *HLA-G5* and *RREB1* mRNA by Quantitative Polymerase Chain Reaction

To study the relative expression of *HLA-G5* and *RREB1*, cDNA synthesis was performed from total RNA using the enzyme M-MLV-RT 200 U/µL (Invitrogen, Carlsbad, California, USA) and SimpliAmp Thermal Cycler equipment (Applied Biosystems). *HLA-G5* primers have been described in Gomes et al. (2018), and they were designed to target all *RREB1* isoforms (RREB-1F: 5′-AAA​GAT​GGT​AGA​AGA​CGG​G-3′ and RREB-1R: 5′-GTG​GGT​TAT​CTG​AAT​GGG​TC-3′). Expression was performed using the SYBR Green DNA intercalator (Applied Biosystems).

### Statistical Analysis

The normality distribution of the miRNA–mRNA expression was determined using the Shapiro–Wilk and Kolmogorov–Smirnov tests. For comparison between two or three groups, the Mann–Whitney U and Kruskal–Wallis tests were used, respectively. A Spearman’s correlation coefficient analysis was performed between miRNA and mRNA expressions. In different experiments, the number of samples may have differed due to the shortage of clinical samples that did not allow all analyses to be performed. The GraphPad Prism V.5.01 (GraphPad Software, Inc.) was used to perform the analyses, considering a significant *p*-value ≤ 0.05. For miRNA relative expression analysis, ∆Ct (cycle threshold) values were determined based on the following equation: ∆Ct = Ct (target miRNA) − Ct (reference miRNA). The Ct values were the average duplicates with a standard deviation (SD) ≤ 0.5. The same equation and parameters were used to calculate the mRNA expression, considering *HLA-G5* or *RREB1* as the target gene and *GAPDH* as the reference gene.

## Results

### Soluble HLA-G Levels in Pediatric Acute Leukemia Patients

**FIGURE 1:**
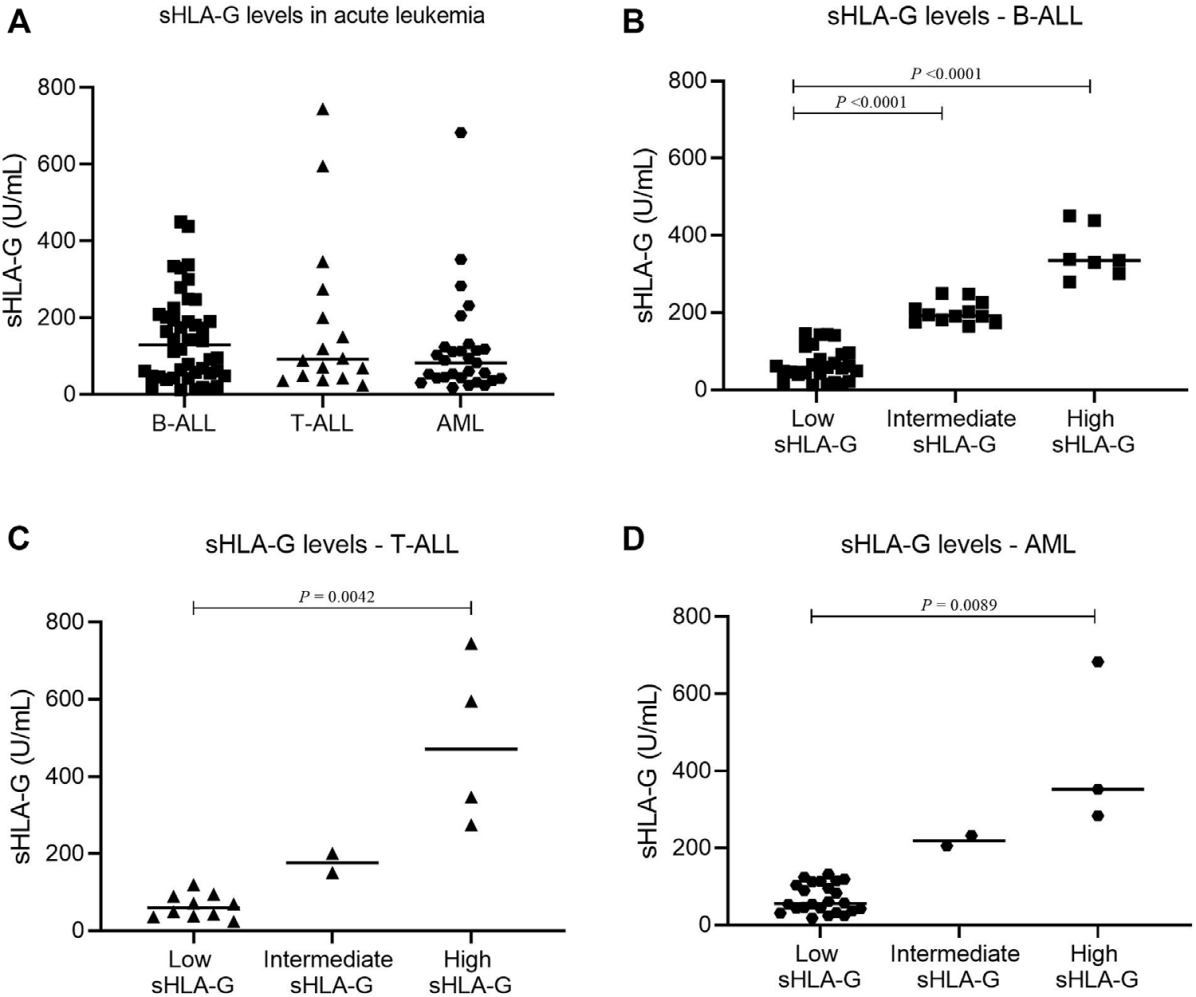
Comparison of sHLA-G levels in the bone marrow stroma of pediatric acute leukemia. **(A)** sHLA-G in B-ALL (square, *n* = 46), T-ALL (triangle, *n* = 16), and AML (hexagon, *n* = 29); **(B)** sHLA-G levels in B-ALL (square: low, *n* = 27; intermediate, *n* = 12; high, *n* = 7); **(C)** sHLA-G levels in T-ALL (triangle: low, *n* = 10; intermediate, *n* = 2; high, *n* = 4); and **(D)** sHLA-G levels in AML (hexagon: low, *n* = 24; intermediate, *n* = 2; high, *n* = 3). For comparison of the three groups, the Kruskal–Wallis test was used followed by Dunn’s multiple comparison for two groups.

Bone marrow sHLA-G levels in childhood AML, T-ALL, and B-ALL showed no statistical differences (*p* = 0.3483). There were low, intermediate, and high sHLA-G producers in each leukemia subtype (Figure 1).

### Identification of Cellular MicroRNAs Upregulated in High Marrow sHLA-G Producers

The analysis of differentially expressed miRNA profiles in the bone marrow cells of non-treated children with ALL revealed 10 miRNAs upregulated in high sHLA-G producers (logFC >2.0) when compared with low sHLA-G producers (Table 2).

**TABLE 2:** miRNAs differentially expressed between childhood ALL high and low soluble HLA-G producers with FDR ≤0.05.

miRNA	LogFC	FDR
**Upregulated in high sHLA-G producers**		
hsa-miR-1248	5.427	0.006
hsa-miR-205-5p	5.870	0.014
hsa-miR-3196	3.824	0.035
hsa-miR-4485-3p	5.380	0.006
hsa-miR-4488	5.036	0.013
hsa-miR-4516	3.560	0.028
hsa-miR-451a	3.003	0.014
hsa-miR-4532	3.846	0.014
hsa-miR-486-5p	2.910	0.014
hsa-miR-5096	2.589	0.030

Note: LogFC, fold change in base 2 logarithm; FDR, false discovery rate.

### Target Prediction of Differentially Expressed MicroRNAs and Functional Annotation

**FIGURE 2:**
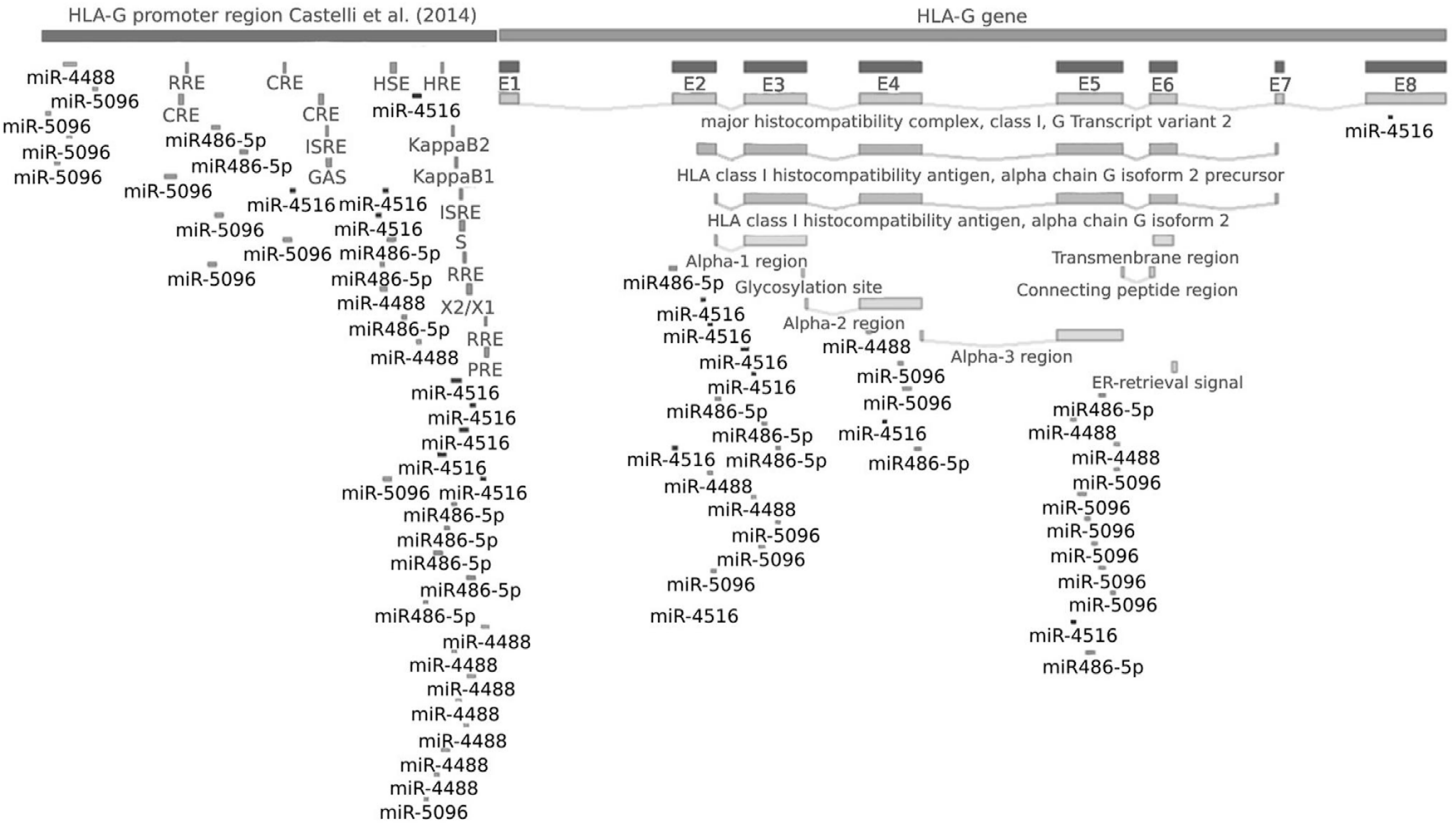
*HLA-G* gene binding sites for hsa-miR-5096, hsa-miR-4516, hsa-miR-486-5p, and hsa-miR-4488. The miRNA cascade in the figure indicates putative binding sites in the target gene. Note: the promoter region was mapped and analyzed as described by Castelli et al. (2014). Coding sequence was considered as described by GenBank (<https:// www.ncbi.nlm.nih.gov/nuccore/NG_029039.1>), and exon 8 is considered as the *HLA-G* 3′UTR [5].

The analysis of target gene prediction with the 10 miRNAs showed 14,518 potential gene targets, of which only the hsa-miR-5096 was predicted as a putative regulator of *HLA-G* mRNA by three different algorithms and, by less number, the hsa-miR-4516, hsa-miR-4488, and hsa-miR-486-5p miRNAs (Figure 2). All four miRNAs presented several anchor sites at the promoter and coding regions of the *HLA-G* gene. Some of the binding sites of miRNAs are in transcription factor zones. The cAMP-responsive element (CRE) is a predicted site for hsa-miR-5096 binding; the heat shock element (HSE) for hsa-miR-486-5p; the hypoxia-responsive element (HRE) for hsa-miR-4516 and hsa-miR-4488; the Kappa B1, Kappa B2 (NF-κB responsive element), interferon-stimulated response element (ISRE) module, and the SXY module for hsa-miR-4488, hsa-miR-4516, and hsa-miR-486-5p; and the Ras-responsive element (RRE) and progesterone-responsive element (PRE) for hsa-miRNA-4516 and hsa-miR-4488. The hsa-miR-5096 did not bind to any of these transcription-binding sites. Only the hsa-miR-4516 targets the *HLA-G* 3′ untranslated region at the position covering the +3035 C/T polymorphic site.

The analysis of the miRNA gene targets for functional annotation revealed several biological pathways involving genes already described in the literature that may be positive or negative regulators for the *HLA-G* gene. Table 3 shows the genes involved in the induction or repression of *HLA-G* transcription and their putative miRNA regulators identified in this study.

**TABLE 3:** Positive and negative regulators of *HLA-G* expression potentially targeted by the DE-miRNAs in childhood ALL, encompassing high marrow sHLA-G producers.

	miR-1248	miR-205-5p	miR-3196	miR-4488	miR-4516	miR-451a	miR-4532	miR-486-5p	miR-5096
**Positive regulators**									
*CREB1*	X	X	—	—	X	—	—	—	X
*CREBBP*	X	—	X	X	—	—	—	—	X
*JUN*	—	X	—	X	—	—	—	—	—
*ATF2*	X	X	—	—	—	X	—	X	X
*IRF1*	X	X	—	—	—	—	X	—	X
*HIF1A*	X	—	—	—	—	—	—	—	—
*IL10*	—	—	—	—	—	—	—	—	X
**Negative regulators**									
*RREB-1*	X	X	X	X	X	—	—	X	X
*HDAC1*	X	—	—	—	X	—	—	—	X
*CTBP1/2*	X	X	X	—	X	—	X	X*	X*
*REST*	X	X	—	—	X	—	—	X	X
*EHMT1*	—	—	X	X	—	—	—	—	—
*ZEB1/2*	X	X	—	—	X	—	—	X**	X**
*ZnF217*	—	—	—	—	X	—	—	X	X

Note: * only *CTBP2* was a target of miR-486-5p and miR-5096. ** miR-486-5p putative targets only *ZEB1*, and miR-5096 targets only *ZEB2*. The hsa-miR-4485-3p was not included in the table because it does not target any of the *HLA-G* regulators above.

**FIGURE 3:**
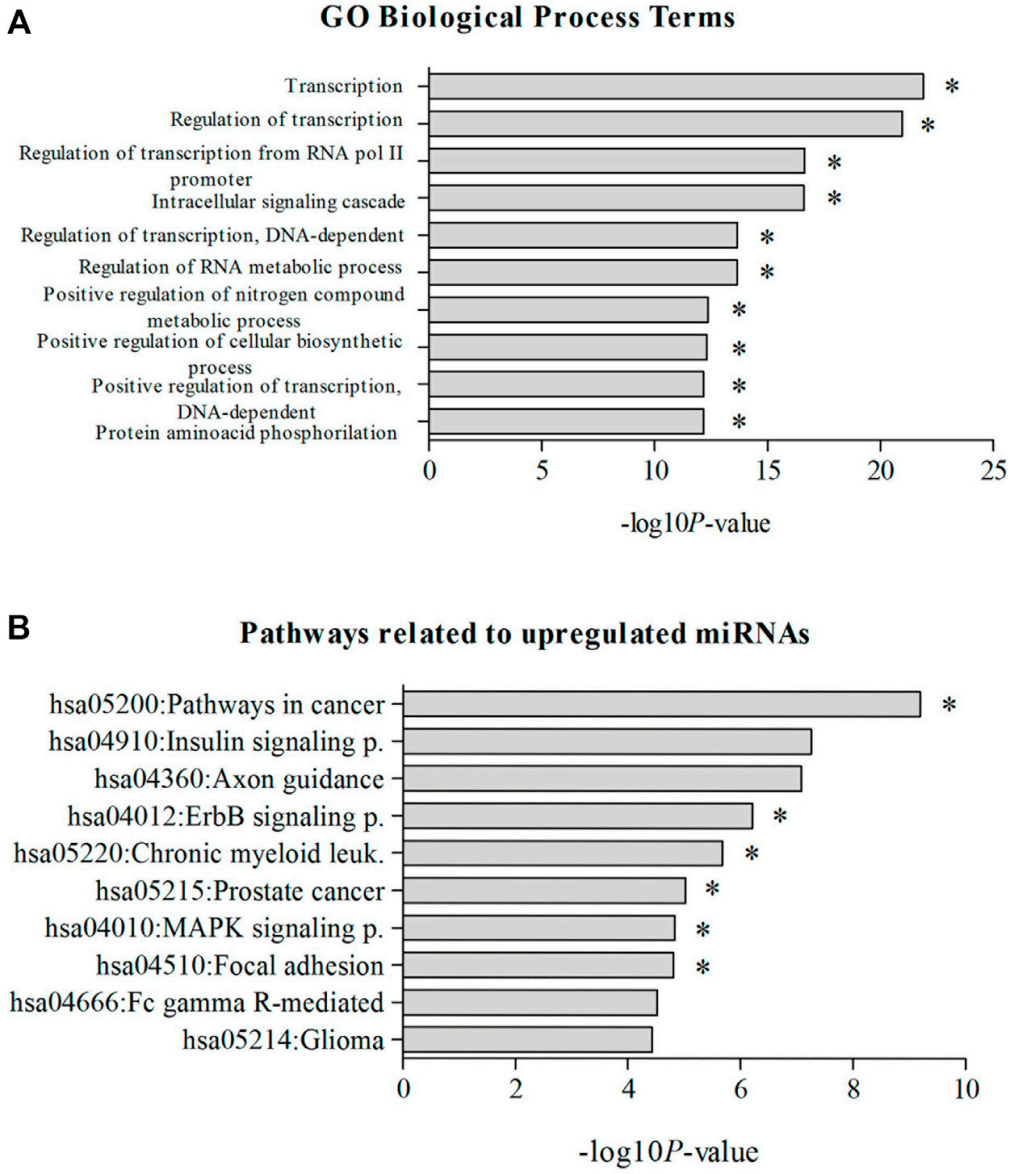
Most significant KEGG pathways. **(A)** GO, biological process terms; **(B)** related to upregulated miRNAs in childhood in ALL patients with high sHLA-G levels. Note: *pathways containing genes coding for positive or negative regulators of *HLA-G* expression. KEGG pathway categories: hsa05200:Pathways in cancer, hsa04910:Insulin signaling pathway, hsa04360:Axon guidance, hsa04012:ErbB signaling pathway, hsa05220:Chronic myeloid leukemia, hsa05215:Prostate cancer, hsa04010:MAPK signaling pathway, hsa04510:Focal adhesion, hsa04666:Fc gamma R–mediated phagocytosis, hsa05214:Glioma. GO, biological process terms: GO:0006350—transcription, GO:0045449—regulation of transcription, GO:0006357—regulation of transcription from RNA polymerase II promoter, GO:0007242—intracellular signaling cascade, GO:0006355—regulation of transcription, DNA dependent, GO:0051252—regulation of RNA metabolic process, GO:0051173—positive regulation of nitrogen compound metabolic process, GO:0031328—positive regulation of cellular biosynthetic process, GO:0045893—positive regulation of transcription, DNA dependent, GO:0006468—protein amino acid phosphorylation.

Considering the 10 most significant KEGG pathways related to all upregulated miRNAs in high sHLA-G producers, 6 included the genes encoding known positive (*CREB1*, *CREBBP*, *JUN*, and *IL10*) and negative (*CTBP1/2* and *HDAC1*) regulators of *HLA-G* expression (Figure 3), as well in other pathways associated with leukemogenesis, namely, hsa04310:Wnt, hsa04660:T-cell receptor, hsa04062:chemokine, hsa04662:B-cell receptor, hsa04330:Notch, and hsa04350:TGF-beta signaling pathways. Most of the statistically significant GO biological processes involved in regulating transcription and cell signaling cascade include inducers (*CREB1*, *CREBBP*, *ATF2*, *JUN*, and *IL10*) and repressors (*RREB1*, *CTBP1/2*, and *HDAC1*) of the *HLA-G* expression (Figure 3).

### Confirmation of Bone Marrow MicroRNA Expression in Childhood Leukemia

**FIGURE 4:**
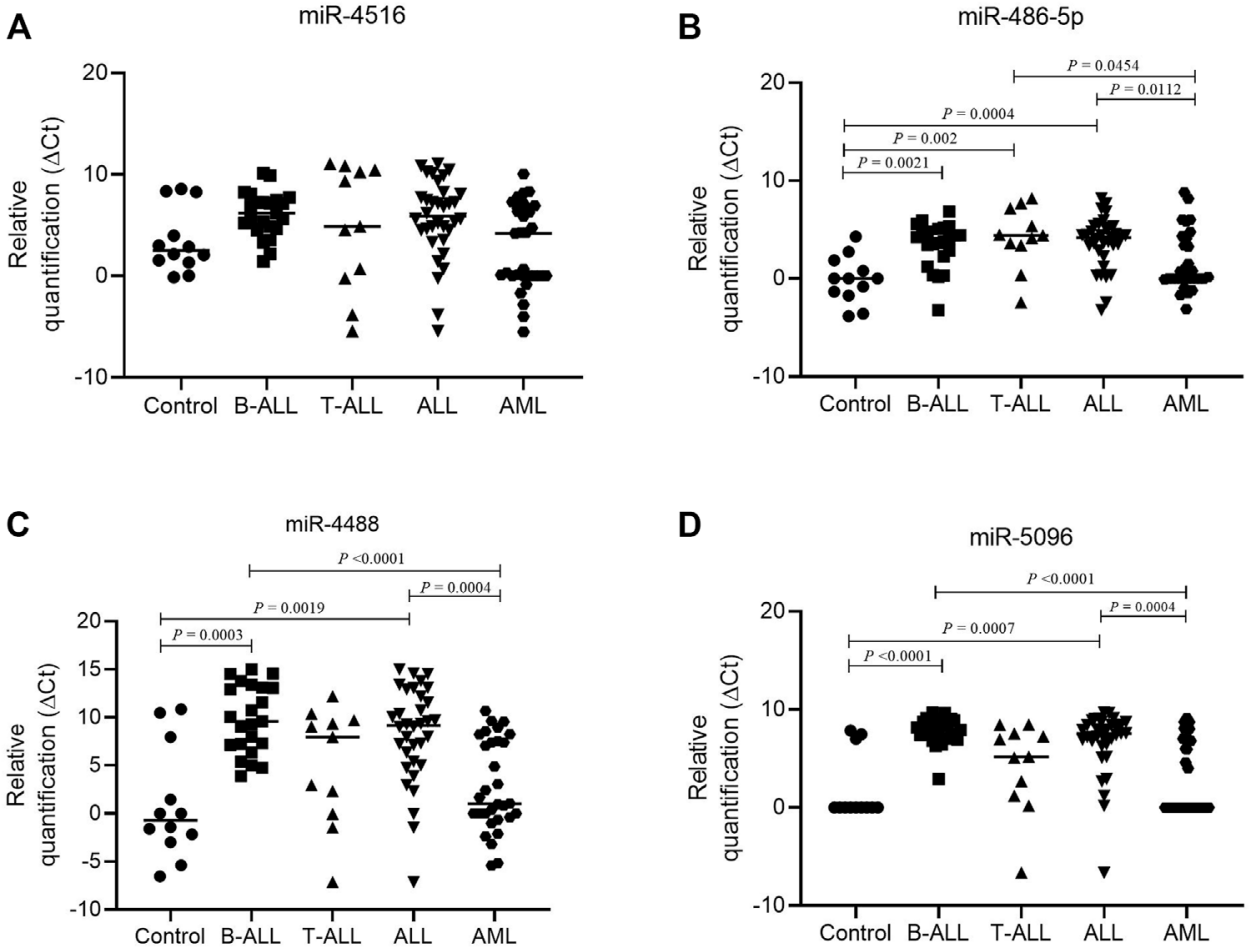
Difference in miRNA expression in lymphoid and myeloid leukemia. **(A)** Relative expression of hsa-miR-4516 in control (circle, *n* = 12), B-ALL (square, *n* = 23), T-ALL (triangle, n = 11), ALL (inverted triangle, *n* = 34), and AML (hexagon, *n* = 31) groups; **(B)** relative expression of hsa-miR-486-5p in control (circle, *n* = 12), B-ALL (square, *n* = 23), T-ALL (triangle, *n* = 11), ALL (inverted triangle, *n* = 34), and AML (hexagon, *n* = 31) groups; **(C)** relative expression of hsa-miR-4488 in control (circle, *n* = 12), B-ALL (square, *n* = 23), T-ALL (triangle, *n* = 11), ALL (inverted triangle, *n* = 34), and AML (hexagon, *n* = 31) groups; and **(D)** relative expression of hsa-miR-5096 in control (circle, *n* = 12), B-ALL (square, *n* = 23), T-ALL (triangle, *n* = 11), ALL (inverted triangle, *n* = 34), and AML (hexagon, *n* = 31) groups. For comparing three or more groups, the Kruskal–Wallis test was used followed by Dunn’s multiple comparison for two groups. Note: For delta Ct, the higher the values, the lower the miRNA expression.

The comparison of the miRNA levels in the bone marrow showed that miR-486-5p, miR-4488, and miR-5096 levels were significantly higher in controls than in ALL, particularly B-ALL, and only miR-486-5p was higher in controls than in T-ALL (*p* < 0.05). No significant differences in miRNA levels were observed between controls and AML bone marrow samples (*p* > 0.05). In addition, the AML samples showed higher miR-4488 and miR-5096 levels than did B-ALL and higher miR-486-5p levels than did T-ALL (*p* < 0.05) (Figure 4).

### Correlations Between MicroRNAs and *HLA-G5* mRNA Levels

**FIGURE 5:**
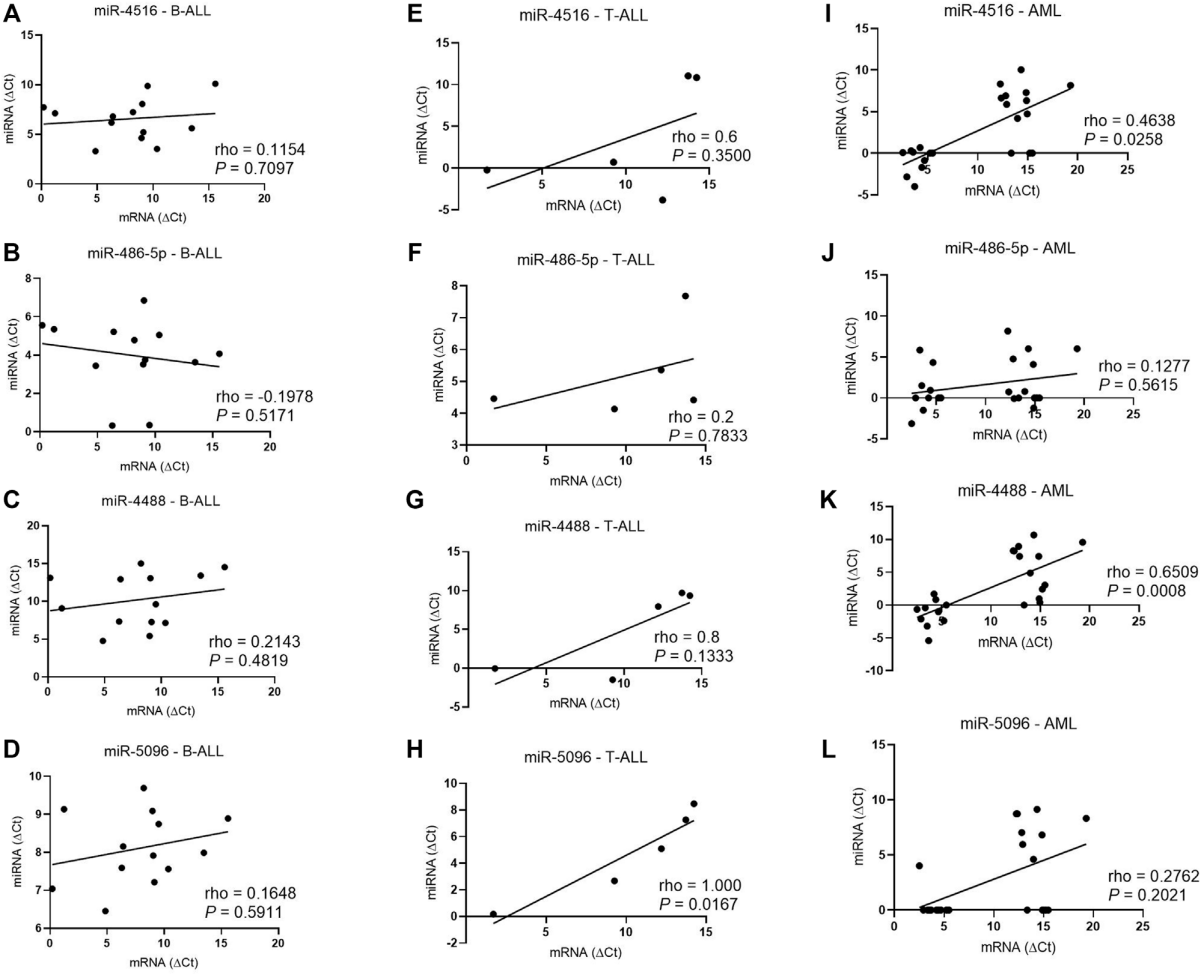
Correlation coefficient analysis between miRNAs and HLA-G5 mRNA levels in the bone marrow from patients with untreated leukemia. **(A)** Correlation coefficient analysis between hsa-miR-4516 and HLA-G5 in B-ALL (*n* = 13); **(B)** correlation coefficient analysis between hsa-miR-486-5p and HLA-G5 in B-ALL (*n* = 13); **(C)** correlation coefficient analysis between hsa-miR-4488 and HLA-G5 in B-ALL (*n* = 13); **(D)** correlation coefficient analysis between hsa-miR-5096 and HLA-G5 in B-ALL (*n* = 13); **(E)** correlation coefficient analysis between hsa-miR-4516 and HLA-G5 in T-ALL (*n* = 5); **(F)** correlation coefficient analysis between hsa-miR-486-5p and HLA-G5 in T-ALL (*n* = 5); **(G)** correlation coefficient analysis between hsa-miR-4488 and HLA-G5 in T-ALL (*n* = 5); **(H)** correlation coefficient analysis between hsa-miR-5096 and HLA-G5 in T-ALL (*n* = 5); **(I)** correlation coefficient analysis between hsa-miR-4516 and HLA-G5 in AML (*n* = 23); **(J)** correlation coefficient analysis between hsa-miR-486-5p and HLA-G5 in AML (*n* = 23); **(K)** correlation coefficient analysis between hsa-miR-4488 and HLA-G5 in AML (*n* = 23); and **(L)** correlation coefficient analysis between hsa-miR-5096 and HLA-G5 in AML (*n* = 23). For the correlation analysis, the Spearman’s correlation coefficient was used.

**FIGURE 6:**
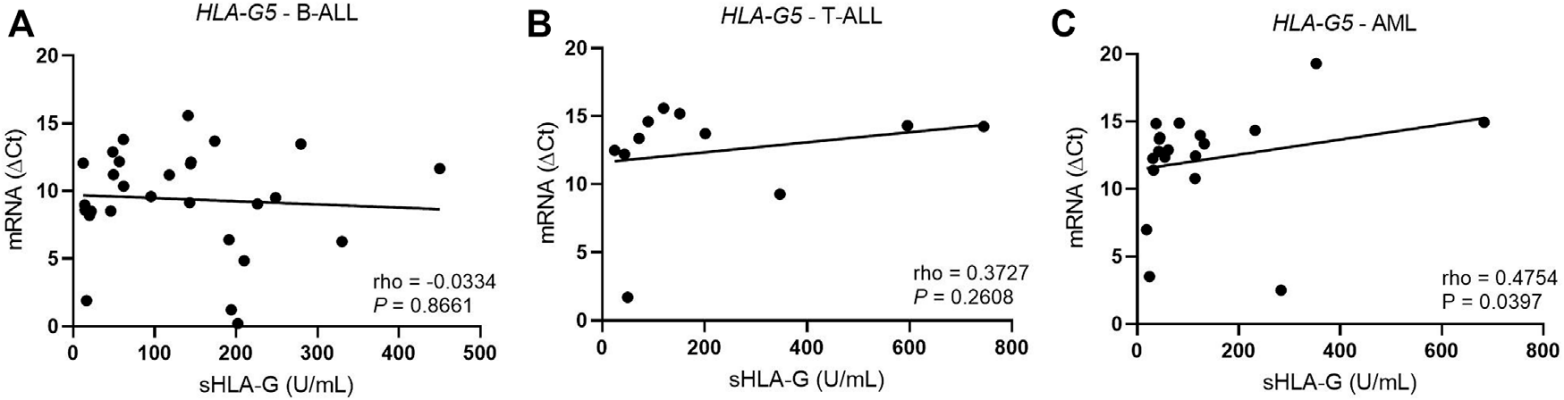
Correlation coefficient analysis between HLA-G5 mRNA and sHLA-G levels in the bone marrow from patients with untreated leukemia. **(A)** Correlation coefficient analysis between HLA-G5 and sHLA-G in B-ALL (*n* = 28); **(B)** correlation coefficient analysis between HLA-G5 and sHLA-G in T-ALL (*n* = 11); and **(C)** correlation coefficient analysis between HLA-G5 and sHLA-G in AML (*n* = 19). For the correlation analysis, the Spearman’s correlation coefficient was used.

In T-ALL, the hsa-miR-5096 levels correlated positively with the *HLA-G5* mRNA expression (rho = 1; *p* = 0.0167) (Figure 5). In myeloid leukemia, the hsa-miR-4516 (rho = 0.4638; *p* = 0.0258) and hsa-miR-4488 (rho = 0.6509, *p* = 0.0008) levels were also positively correlated with the *HLA-G5* mRNA levels. However, the increase in *HLA-G5* mRNA expression was translated into a significant decrease in sHLA-G only in myeloid leukemia, with moderate and significant Spearman’s coefficient (rho = 0.475; *p* = 0.0397), but neither in B-ALL nor T-ALL (Figure 6). This was assumed considering that the delta Ct values are inversely proportional to the mRNA levels.

**FIGURE 7:**
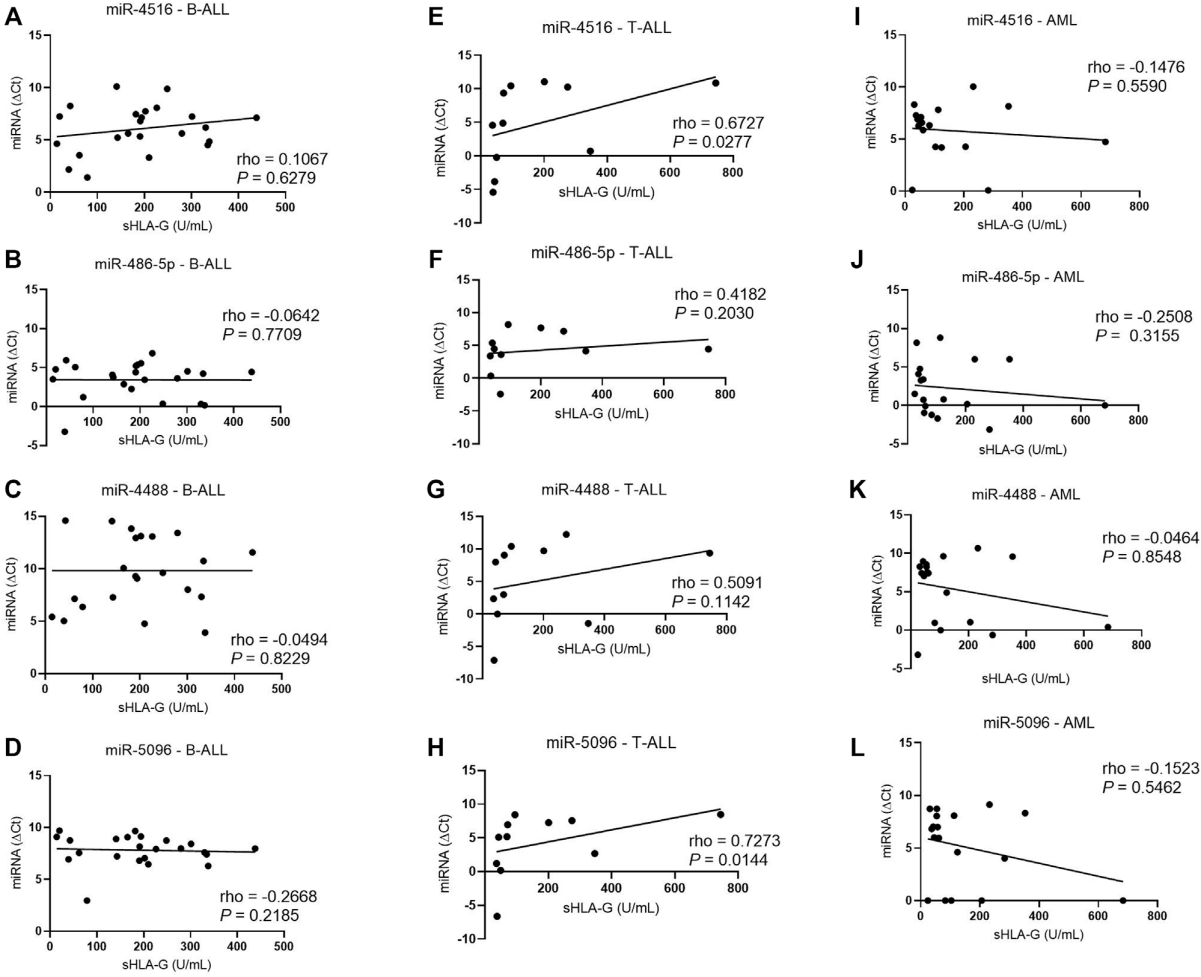
Correlation coefficient analysis between miRNAs expression and sHLA-G levels in the bone marrow from patients with untreated leukemia. **(A)** Correlation between hsa-miR-4516 and sHLA-G in B-ALL (*n* = 23); **(B)** correlation between hsa-miR-486-5p and sHLA-G in B-ALL (*n* = 23); **(C)** correlation between hsa-miR-4488 and sHLA-G in B-ALL (*n* = 23); **(D)** correlation between hsa-miR-5096 and sHLA-G in B-ALL (*n* = 23); **(E)** correlation between hsa-miR-4516 and sHLA-G in T-ALL (*n* = 11); **(F)** correlation between hsa-miR-486-5p and sHLA-G in T-ALL (*n* = 11); **(G)** correlation between hsa-miR-4488 and sHLA-G in T-ALL (*n* = 11); **(H)** correlation between hsa-miR-5096 and sHLA-G in T-ALL (*n* = 11); **(I)** correlation between hsa-miR-4516 and sHLA-G in AML (*n* = 18); **(J)** correlation between hsa-miR-486-5p and sHLA-G in AML (*n* = 18); **(K)** correlation between hsa-miR-4488 and sHLA-G in AML (*n* = 18); and **(L)** correlation between hsa-miR-5096 and sHLA-G in AML (*n* = 18). For the correlation analysis, the Spearman’s correlation coefficient was used.

In addition, increased hsa-miR-5096 (rho = 0.72; *p* = 0.0144) and hsa-miR-4516 (rho = 0.67; *p* = 0.0277) levels (low ΔCt) correlated with decreased sHLA-G protein levels in T-ALL, but only hsa-miR-5096 correlated also with the HLA-G mRNA (Figure 7), but only hsa-miR-5096 correlated also with the *HLA-G* mRNA.

**FIGURE 8:**
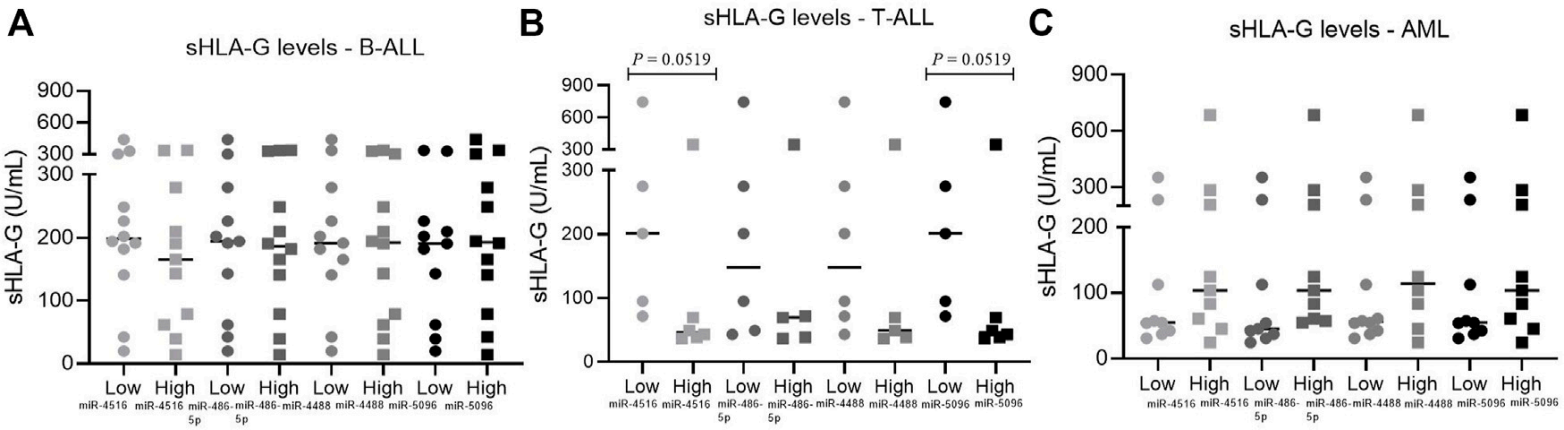
Relationship between sHLA-G with miRNAs expression in leukemic bone marrow. **(A)** Relationship between sHLA-G and miRNAs expression in B-ALL: low miR-4516, *n* = 12; high miR-4516, *n* = 11; low miR-486-5p, *n* = 11, high miR-486-5p, *n* = 12; low miR-4488, *n* = 11, high miR-4488, *n* = 12; low miR-5096, *n* = 11, high miR-5096, *n* = 12; **(B)** relationship between sHLA-G and miRNAs expression in T-ALL: low miR-4516, *n* = 5, high miR-4516, *n* = 6; low miR-486-5p, *n* = 6, high miR-486-5p, *n* = 5; low miR-4488, *n* = 6, high miR-4488, *n* = 5; low miR-5096, *n* = 5, high miR-5096, *n* = 6; **(C)** relationship between sHLA-G and miRNAs expression in AML: low miR-4516, *n* = 9, high miR-4516, *n* = 9; low miR-486-5p, *n* = 9, high miR-486-5p, *n* = 9; low miR-4488, *n* = 10, high miR-4488, *n* = 8; low miR-5096, *n* = 9, high miR-5096, *n* = 9. For comparison of two groups, the Mann–Whitney test was used.

For a detailed analysis, the samples were categorized according to the bone marrow miRNA levels in the low or high miRNA level group, and sHLA-G levels in both groups were compared. In T-ALL, patients with high levels of hsa-miR-5096 and miR-4516 had a median sHLA-G value of 46 U/mL, while patients with low levels of miRNA had a median sHLA-G value of 200 U/mL (*p* = 0.0519). Overall, high miRNA expressions were associated with homogenous low sHLA-G levels, while low miRNA levels were associated with largely variable sHLA-G levels, which contributed to the borderline significance of the differences. In B-ALL, the groups of low and high miRNA levels were not capable of segregating samples with different sHLA-G levels. In AML, the difference between the median value of sHLA-G between the low- and high-miRNA-level groups was not significant (Figure 8).

Considering that the *RREB1* gene is a target for the four studied miRNAs and that the RREB-1 protein has three potential binding sites in the *HLA-G* gene promoter, the relationship between the *RREB1* mRNA levels and each miRNA and *HLA-G5* mRNA levels were evaluated. The results revealed that only in B-ALL, the *RREB1* and *HLA-G5* mRNA expressions were positively correlated (rho = 0.5632, *p* = 0.0018). In addition, only the hsa-miR-4488 correlated positively with *RREB1* mRNA expression (rho = 0.4368, *p* = 0.0615), but it did not reach significance.

## Discussion

In this study, the evaluation of the differential expression profiles of miRNAs in the bone marrow among leukemia patients exhibiting high and low marrow sHLA-G levels envisaged the identification of new regulators of *HLA-G* that may play a role in cancer immunosurveillance (Castelli et al., 2014; Lin and Yan, 2018; Aguagué et al., 2011; Paul et al., 1998). Few or no studies have reported many of the 10 differentially expressed miRNAs as modulators of HLA-G expression. Interestingly, according to the next-generation sequencing analysis, all miRNAs were upregulated in the group of high HLA-G producers, suggesting that these miRNAs target the *HLA-G* gene sequence; however, they do not downregulate *HLA-G* expression. Previous studies focusing on the *TNF* gene (Vasudevan et al., 2007) have shown alternative mechanisms of action of miRNAs, increasing the transcription of the target gene and the expression of target proteins, dependent on the micro-ribonucleoproteins (microRNPs) and gene regions (promoter or coding region), with which miRNAs interact (Vasudevan et al., 2007; Place et al., 2008). The sequence alignment analysis showed that the hsa-miR-5096, hsa-miR-4516, hsa-miR-4488, and hsa-miR-486-5p miRNAs are capable of binding multiple sites at coding and 5′ untranslated region of the *HLA-G* gene and a unique binding site for hsa-miR-4516 at the 3′ untranslated region.

The validation experiments showed that the hsa-miR-4516 levels in the bone marrow did not differ significantly in non-leukemic and leukemia samples. However, the relationship between the high hsa-miR-4516 levels and low sHLA-G protein levels in the bone marrow in T-ALL indicated that the classic mechanism of negative regulation by the miRNA exerted at the 3′UTR of the *HLA-G* gene was active. The correlation coefficient analysis revealed that increased hsa-miR-4516 levels (low delta Ct values) correlated with lower sHLA-G levels. Previously, a study reported the hsa-miR-4516 as a potential regulator of *HLA-G* expression based on *in silico* study, which showed a putative binding between the two molecules but lacked functional studies (Porto et al., 2015). The predicted interaction between hsa-miR-4516 and *HLA-G* occurs at the +3035 polymorphic site of 3′UTR of the *HLA-G* gene, which might affect the hsa-miR-4516–mediated downregulation of *HLA-G* expression. In T-ALL, we also observed that increased hsa-miR-5096 expression levels correlated positively with *HLA-G5* mRNA and negatively with sHLA-G levels. One of the predicted binding sites for the hsa-miR-5096 is the CRE site at the *HLA-G* promoter, which induces gene transcription in response to cAMP (Gobin et al., 2002). The hsa-miR-5096 was reported as a potential tumor suppressor miRNA capable of inhibiting the proliferation, migration, and invasion of breast cancer cells *in vitro* by targeting the SLC7A11 gene, which is related to ferroptosis resistance (Yadav et al., 2021). On the other hand, Thuringer et al. (2017) demonstrated an oncogene role for hsa-miR-5096 whose high expression contributed to increased invasiveness of glioblastoma cells by decreasing Kir4.1 protein levels, a K+ channel involved in the ionic homeostasis in the brain (Thuringer et al., 2017). The hsa-miR-5096 seems to target different genes in distinct cell types and microenvironments with a different action mechanism, which may occur also in leukemia. Similarly, the cell heterogeneity could partially explain the difference between miRNA sequencing results and qPCR experiments. In addition, it should be considered that the sHLA-G protein levels depend on the resultant effect of the negative and positive regulators of the *HLA-G* expression, the own expression of which is regulated by hsa-miR-5096 and hsa-miR-4516.

In B-ALL, we observed a moderate correlation between the *HLA-G* expression and one of its negative regulators, the RREB-1, and apparently, the *RREB1* expression correlated with hsa-miR-4488 levels in the bone marrow. The RREB-1 protein is a well-known repressor of *HLA-G* expression, interacting with the *HLA-G* gene at three different sites in the promoter region (Flajollet et al., 2009). In addition, it is important to note that RREB-1 acts in a complex with other proteins, HDAC1, CtBP1/2, REST, EHMT1, ZEB1/2, and ZnF217, which are involved in chromatin remodeling and transcription machinery assembly (Delcuve et al., 2012; Barroilhet et al., 2013; Ray et al., 2014; Vitkevičienė et al., 2019) and are also targets for these differentially expressed miRNAs. However, the role of hsa-miR-4488 at the RRE site is unclear, since hsa-miR-4488 regulates the expression of RREB1, which induces the downregulation of *HLA-G* expression by binding to the RRE site. Further studies evaluating the role of miRNA/*RREB1*/*HLA-G* interaction may clarify whether hsa-miR-4488 competes with the RREB-1 factor for the RRE site at the *HLA-G* promoter. Hsa-miR-4488 has been reported with aberrant expression in other cancers, such as colorectal cancer (Zhang et al., 2014) and melanoma (Fattore et al., 2019), and its increased expression has been associated with drug resistance in melanoma cell lines (Fattore et al., 2019). To associate the high hsa-miR-4488 levels in the bone marrow with chemotherapy resistance in T-ALL, a larger casuistic would be necessary.

In AML, the hsa-miR-4488, hsa-miR-486-5p, and hsa-miR-5096 levels in the bone marrow were higher than were in ALL, hsa-miR-4488 and hsa-miR-4516 expressions correlated with *HLA-G5* expression (*p* = 0.0008 and *p* = 0.0258, respectively), and the increased *HLA-G5* expression correlated with low sHLA-G levels, but no miRNA expression correlated with the sHLA-G levels.

Previous studies of extracellular vesicles from breast cancer cells reported that hsa-miR-4488 was negatively correlated to the mitochondrial calcium uniporter and that was related to the suppression of angiogenesis of vascular endothelial cells by acting on *CX3CL1*. Its absence or absent expression appeared to increase angiogenesis and favor metastasis in breast cancer cells (Zheng et al., 2020). This study was the first to report the effect of hsa-miR-4488 in hematological cancer, with a significantly less hsa-miR-4488 level in AML and a much lesser one in ALL when compared to the non-leukemic bone marrow. The hsa-miR-4488 mechanism of action in physiologic and pathologic bone marrow remains unknown.

Besides the high levels of hsa-miR-486-5p in AML when compared to ALL, there was no significant difference between the AML levels and non-leukemic bone marrow levels. The higher miRNA level in non-leukemic bone marrow corroborates the function of hsa-miR-486-5p in the induction of growth and survival of megakaryocyte–erythroid progenitors (Wang et al., 2015). The hsa-miR-486-5p level was reported to be downregulated in the peripheral blood leukocytes in untreated chronic myeloid leukemia (CML) adult patients, which was upregulated after imatinib treatment (Ninawe et al., 2021). Another study showed that high miR-486-5p levels induced apoptosis and caspase-3 activity in leukemic cells by upregulating the *FOXO1* mRNA expression (Liu et al., 2019). On the contrary, another study suggested that hsa-miR-486-5p might be involved in the growth and survival of leukemic cells in AML secondary to Down syndrome, which generally compromises the megakaryocyte–erythroid precursors (Wang et al., 2015). Our casuistries were of children with leukemia, and cases of CML are rare; therefore, the mechanism of action of hsa-miR-486-5p in ALL remains unclear. Is it associated with the reduced number of megakaryocyte–erythroid progenitors observed?

Nine of 10 hsa-miRNAs revealed in this study, namely, hsa-miR-1248, hsa-miR-205-5p, hsa-miR-3196, hsa-miR-4488, hsa-miR-4516, hsa-miR-451a, hsa-miR-4532, hsa-miR-486-5p, and hsa-miR-5096, exhibited the ability to interact with at least one gene (*CREB1*, *CREBBP*, *JUN*, *ATF2*, *IRF1*, *HIF1A*, and *IL10*) coding for a protein involved in the induction of *HLA-G* expression. Moreover, the CREB1, CREBBP, C-Jun, ATF-2, IRF-1, and HIF-1A are well-known proteins that bind to specific promoter sites of the *HLA-G* gene activating its transcription (Gobin et al., 2002; Mouillot et al., 2007; Castelli et al., 2014; Garziera et al., 2017). Soluble mediators, such as IL-10, IFN-β, and IFN-γ cytokines and progesterone hormone, are capable of inducing *HLA-G* expression *via* intracellular signaling pathway; therefore, a possible interaction between miRNAs mentioned above in these mediators’ genes can also decrease the *HLA-G* expression (Moreau et al., 1999; Chu et al., 1999; Lefebvre et al., 1999; Yie et al., 2006).

The resulting effect of all variables directly or indirectly involved in the *HLA-G* expression in physiological and pathological bone marrow is not yet known. Our study added new information on the regulation of HLA-G levels in leukemia. We identified four new miRNA molecules associated with the *HLA-G* expression regulation and its predicted target genes. We showed that some miRNA and target gene levels correlated with the *HLA-G* mRNA and protein levels in the tumor microenvironment. We also showed that the miRNA expression and regulation differed according to the leukemia type.

Future studies in a more extensive series of patients could support the hypothesis that miRNAs’ regulation of sHLA-G expression may play a role in the prognosis of acute leukemias, indicating the potential translation of these results in clinical practice, possibly as a new prognosis marker and target for immunotherapy.

## Data Availability

The data presented in the study are deposited in the ArrayExpress database at EMBL-EBI (www.ebi.ac.uk/arrayexpress) repository, accession number E-MTAB-11621.

## Abbreviations

3′UTR, 3′ untranslated region; 5′ UTR, 5′ untranslated region; ALL, acute lymphoblastic leukemia; AML, acute myeloid leukemia; B-ALL, acute lymphoblastic leukemia of B cells; cDNA, complementary DNA; CRE, cAMP response element–binding protein; Ct, cycle threshold; DE-miRNA, differentially expressed microRNA; FDR, false discovery rate; HLA-G, human leukocyte antigen G; HRE, hypoxia responsive element; ILT2, Ig-like transcript 2; ILT4, Ig-like transcript 4; ISRE, interferon-stimulated response element; KEGG, Kyoto Encyclopedia of Genes and Genomes; KIR2DL4, killer cell immunoglobulin-like receptor, two Ig domains and long cytoplasmic tail 4; LILRB1, leukocyte immunoglobulin-like receptor B1; LILRB1, leukocyte immunoglobulin-like receptor B2; MHC, major histocompatibility complex; miRNA/miR, microRNA; mRNA, messenger RNA; NK, natural killer cells; PCR, polymerase chain reaction; RREB-1, Ras-responsive element-binding protein 1; RT-PCR, reverse transcription polymerase chain reaction; sHLA-G, soluble human leukocyte antigen G; T CD8, leukocyte T cluster differentiation 8; T-ALL, acute lymphoblastic leukemia of T cells.
